# The Adaptive Olfactory Measure of Threshold (ArOMa-T): a rapid test of olfactory function

**DOI:** 10.1093/chemse/bjac036

**Published:** 2022-12-05

**Authors:** Elisabeth M Weir, Mackenzie E Hannum, Danielle R Reed, Paule V Joseph, Steven D Munger, John E Hayes, Richard C Gerkin

**Affiliations:** Sensory Evaluation Center, College of Agricultural Sciences, The Pennsylvania State University, University Park, PA 16802, United States; Department of Food Science, College of Agricultural Sciences, The Pennsylvania State University, University Park, PA 16802, United States; Monell Chemical Senses Center, Philadelphia, PA 19104, United States; Monell Chemical Senses Center, Philadelphia, PA 19104, United States; Division of Intramural Clinical and Biological Research (DICBR), National Institute on Alcohol Abuse and Alcoholism, National Institutes of Health, Bethesda, MD 20892, United States; Division of Intramural Research, National Institute of Nursing Research, National Institutes of Health, Bethesda, MD 20892, United States; Department of Pharmacology and Therapeutics, College of Medicine, University of Florida, Gainesville, FL 32610, United States; Department of Otolaryngology, College of Medicine, University of Florida, Gainesville, FL 32610, United States; Center for Smell and Taste, University of Florida, Gainesville, FL 32610, United States; Sensory Evaluation Center, College of Agricultural Sciences, The Pennsylvania State University, University Park, PA 16802, United States; Department of Food Science, College of Agricultural Sciences, The Pennsylvania State University, University Park, PA 16802, United States; School of Life Sciences, Arizona State University, Tempe, AZ 85287, United States

**Keywords:** COVID-19, anosmia, detection threshold, adaptive algorithm, sex differences, smell

## Abstract

Many widely used psychophysical olfactory tests have limitations that can create barriers to adoption. For example, tests that measure the ability to identify odors may confound sensory performance with memory recall, verbal ability, and prior experience with the odor. Conversely, classic threshold-based tests avoid these issues, but are labor intensive. Additionally, many commercially available tests are slow and may require a trained administrator, making them impractical for use in situations where time is at a premium or self-administration is required. We tested the performance of the Adaptive Olfactory Measure of Threshold (ArOMa-T)—a novel odor detection threshold test that employs an adaptive Bayesian algorithm paired with a disposable odorant delivery card—in a non-clinical sample of individuals (*n* = 534) at the 2021 Twins Day Festival in Twinsburg, OH. Participants successfully completed the test in under 3 min with a false alarm rate of 7.5% and a test–retest reliability of 0.61. Odor detection thresholds differed by sex (~3.2-fold lower for females) and age (~8.7-fold lower for the youngest versus the oldest age group), consistent with prior studies. In an exploratory analysis, we failed to observe evidence of detection threshold differences between participants who reported a history of COVID-19 and matched controls who did not. We also found evidence for broad-sense heritability of odor detection thresholds. Together, this study suggests the ArOMa-T can determine odor detection thresholds. Additional validation studies are needed to confirm the value of ArOMa-T in clinical or field settings where rapid and portable assessment of olfactory function is needed.

## Introduction

Olfactory dysfunctions are highly prevalent with serious consequences for health, diet, safety, and quality of life (Keller and Malaspina 2013; Boesveldt et al. 2017), including altered diet, a decreased ability to detect dangers such as fire or spoiled food, and poor social connections with other people. Recent estimates suggest up to 1 in 4 people may have some type of olfactory disorder (Parma and Boesveldt 2021). Common causes for olfactory disorders include head trauma, sinonasal disease, and upper respiratory infections (Brämerson et al. 2004; Landis et al. 2005), and prevalence increases with age (Doty 1989; Hummel et al. 2017). Olfactory deficits may also be early biomarkers of neurodegenerative diseases (Gerkin et al. 2017). Despite the impact of olfactory disorders on health and quality of life, olfactory function is infrequently tested outside of research settings (Miman et al. 2011). Public awareness of anosmia increased dramatically in 2020 when sudden smell loss was highlighted as a key symptom of COVID-19 (e.g. Gerkin et al. 2021). Data from initial waves of the pandemic suggested up to ~75% of COVID-19-positive individuals experienced a transient loss of smell (Hannum et al. 2020), and such loss persisted in millions of individuals (Ohla et al. 2022) with substantial impacts on quality of life (Elkholi et al. 2021).

To determine if an individual has a quantitative olfactory disorder, psychophysical testing by a proctor is typically used; such testing can determine if the participant is experiencing anosmia (a complete or near complete loss of smell) or hyposmia (where their ability to perceive odors is substantially reduced but not absent). Notably, quantitative tests are not optimized to assess qualitative disorders like parosmia (distorted smell) or phantosmia (distorted smell), which depend on verbal report. Quantitative tests typically measure 1 or more specific parameters of smell function: odor identification (“what is this? vanilla!”), odor discrimination (“is this smell different from the last one?”), and odor detection threshold (“what is the lowest concentration the person can smell?”).

Two of the most common tests in clinical and research settings are the University of Pennsylvania Smell Identification Test (UPSIT), which is composed of 40 odor identification questions (Doty et al. 1984), and Sniffin’ Sticks, which measures odor identification, odor discrimination, and odor detection threshold (Kobal et al. 1996) to create a composite score or index of function. Within clinical samples, multiple measures of olfactory performance are typically highly, although not fully, correlated (e.g. Cain and Stevens 1989; Doty et al. 1994; Parma and Boesveldt 2021), so using just 1 measure often suffices. A recent meta-analyses of odor identification, odor threshold, and odor discrimination data from Sniffin’ Sticks and the UPSIT confirms the widely held belief that women tend to outperform men, although the authors also caution that effect sizes tend to be small (Sorokowski et al. 2019).

Widely used psychophysical tests for hyposmia and anosmia have some limitations. For example, odor identification tasks require the individual to (i) smell the stimulus, (ii) recognize the stimulus from prior experience, and (iii) communicate the correct name. This sequence confounds functional measurement of sensory performance with memory recall and verbal ability. Also, ability to identify a given odor depends on prior exposure to that stimulus, and this may vary across cultures. For example, root beer is widely known in the United States, but not Europe or Asia. Thus, failure of a European to identify root beer correctly could reflect a failure of familiarity rather than a true sensory impairment per se. Accordingly, odor identification tests must be validated in different populations globally to obtain appropriate normative data (e.g. Oleszkiewicz et al. 2019). Separately, odor identification tests place cognitive demands on participants that may be especially salient for elderly patients or other special populations (e.g. children, or those with cognitive impairments). For odor discrimination tests, functional working memory is required to allow a comparison to be made, and this assumption may not be valid in some populations with limited cognitive function.

By contrast, olfactory tests that measure odor detection threshold have several potential advantages. Such tests avoid issues of familiarity, naming, and recall (Hedner et al. 2010). They also may be more sensitive and/or specific measures of hyposmia and anosmia. That is, the odorants given in an odor identification task are normally presented at a concentration well above threshold, so a small but real drop in olfactory function may be missed (Schubert et al. 2017). In this case, a small drop in perceived intensity may not impair the ability of the patient to successfully identify or name the odor, despite the presence of a true quantitative loss. Still, odor detection threshold tests are used infrequently, in part due to test duration or issues of stimulus control or delivery. Specifically, threshold estimation using traditional methods like an up/down staircase (Cornsweet 1962) can take 30 min or more to get a single measure of threshold. Likewise, traditional odor delivery systems (i.e. plastic squeeze bottles, odor jars, and olfactometers) are cumbersome and are not typically single use items.

Recently, we have developed a novel odor detection threshold test, the Adaptive Olfactory Measure of Threshold (ArOMa-T), which we describe here. This card-based tool is paired with an adaptive algorithm (based on a Bayesian threshold model) that is delivered via an app on a smartphone, tablet, or computer, to rapidly guide users through a task that delivers the odorant concentrations that will be maximally informative in estimating an individual’s odor detection threshold. As an initial proof of concept—i.e. we could create and implement a rapid disposable odor detection threshold test—we deployed the ArOMa-T among a group of individuals without active COVID-19 who were attending the 2-day 2021 Twins Day Festival in Twinsburg, OH.

## Methods

### Participants

The protocol was approved by the local Institutional Review Board for the Monell Chemical Senses Center (IRB protocol#: 843798), and the study followed the principles of the Declaration of Helsinki. Per University of Florida requirements, an additional protocol was approved to receive and analyze anonymized aggregate data (IRB protocol #: IRB202102968). Participants were recruited, consented, and enrolled at a tent managed by the Monell Center in Twinsburg, OH; 595 participants enrolled in the study between 7 and 9 August 2021. All participants provided informed consent electronically. Demographic characteristics of participants (*n* = 534; 29.8% male and 70.2% female; mean age 39.3) are summarized in Table 1. The cohort was predominantly female and White, with a mean age of 39.2 years (± SD of 15.7 years) and a median age of 34.1.

**Table 1.** Participant demographics for ArOMa-T Twins Day study after data cleaning.

Sex	
Male	159
Female	375
Race/ethnicity	
White	459
Black/African American	35
American Indian/Alaska Native	5
Hispanic/Latino	4
Other	6
Prefer not to answer	5
Did not answer	20
Age (mean ± SD) in years	39.2 ± 15.7

### Inclusion and exclusion criteria

**Fig. 1.**
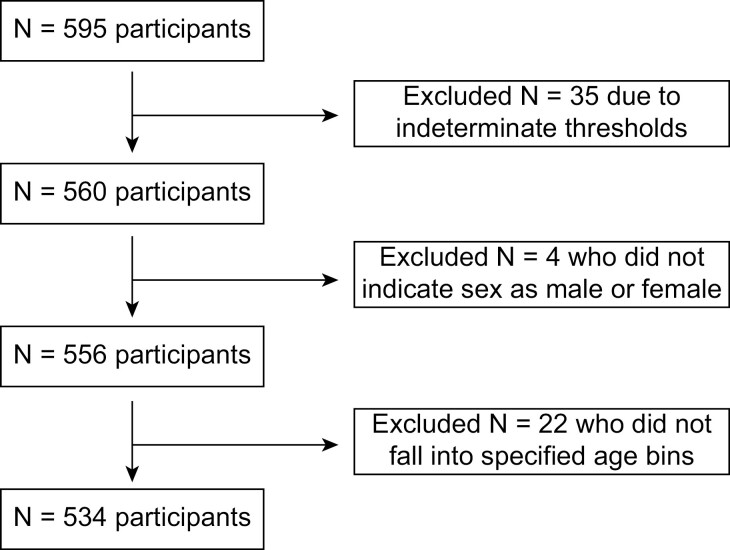
Flow diagram summarizing data cleaning steps resulting in the final participant set used for these analyses (*n* = 534).

Some participants were excluded from the analyses (Fig. 1). Participants (*n* = 35) that had an indeterminate threshold from inconsistent response patterns were excluded. Because a very small number of participants did not self-identify as male or female (*n* = 4), these individuals were excluded from the analysis to allow for models testing sex effects. The participant pool was highly age diverse, so we classified participants into 3 age bins of equal size (in years) for analyses: young (18–37 years), middle aged (38–57 years), and older (58–77). Participants (*n* = 22) who were younger than 18 years or older than 78 years were excluded from the analysis (to facilitate the construction of equally sized age bins). This resulted in a final dataset of 534 unique individuals; a subset of participants (*n* = 97) returned the next day to repeat the test. These data were used to calculate an initial estimate of test–retest reliability using Pearson’s *R* (this was done to ensure that similar results would be seen with repeated testing in a single individual).

### The ArOMa-T

**Fig. 2.**
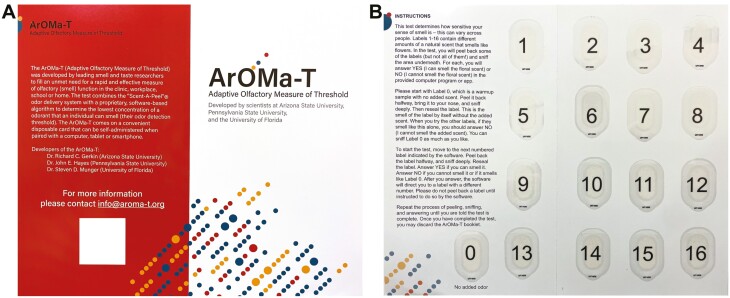
The ArOMa-T. The test includes a bi-fold card graphics and text on the outside pages (A) and with user instruction and 17 peel-and-burst panels that contain varying amounts of the rose-like/floral odorant phenylethyl alcohol on the inside pages (B).

The ArOMa-T, version 1.P8.1, consists of an app paired with a physical card. The bi-fold card (Fig. 2) has graphics and text on the outside face, and the interior face of the folded card has user instructions, along with 17 elliptical Scent-a-Peel peel-and-burst labels (Scentisphere LLC, Carmel, NY) containing an odorant. Similar odorant-release technologies have been used in other tests (e.g. Okutani et al. 2013; Parma et al. 2021). Peel-and-burst delivery systems have several advantages over scratch and sniff encapsulation: (i) because peel-and-burst panels can be resealed, odor delivery can be terminated, and (ii) a peel-and-burst system is more consistent, as the amount of odorant delivered from a scratch and sniff panel varies substantially as a function of the pressure applied and area scratched. The label consists of encapsulated odorant sandwiched between 2 plastic layers. The layers are held together by a ring of adhesive at the periphery. The bottom layer of the label is attached to the card, so that peeling back the top layer releases the odorant. This proprietary technology (from Scentisphere) is also used in the SCENTintel test (Parma et al. 2021). The labels in the ArOMa-T version tested here contain various concentrations of the floral odorant phenylethyl alcohol (PEA). PEA is used widely in smell testing, including in commercial smell tests marketed by Sensonics International (UPSIT, Haddon Heights, NJ) and Burghart GmbH (Sniffin’ Sticks, Holm, Germany). PEA cannot be discriminated by anosmic individuals, as it is not a chemesthetic stimulus (Cometto-Muñiz and Cain 1990). PEA also has abundant normative data in odor detection threshold testing (e.g. Croy et al. 2009), which should facilitate calibration with other assessments.

Each ArOMa-T card contains 3 labels with no odorant, 1 label each of the lowest and highest odorant concentrations, and 2 labels each for the intermediate odorant concentrations (i.e. 8 concentrations in total, excluding the blanks). The concentration range covers ~3.5 orders of magnitude (0.01–30% w/w) and spacing between concentrations are half-log steps (i.e. 0.5 log_10_ units of concentration, or half an order of magnitude). This range was chosen to roughly span the range of normal human detection thresholds for PEA (Oleszkiewicz et al. 2019). The highest concentration contained in the card is arbitrarily denoted as 0 on the log_10_ scale. The unfolded card is approximately 9.5 × 11.15 inches (~241 mm by ~286 mm), and the folded card fits easily within a standard 7 in ×10 in envelope to allow for delivery via mail.

**Fig. 3.**
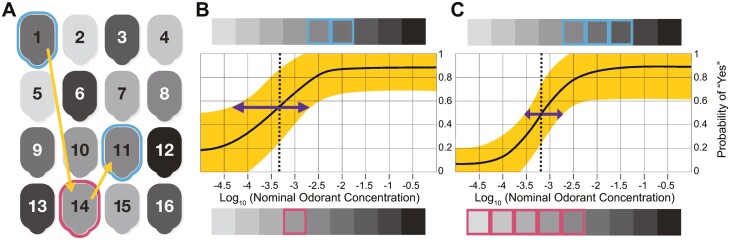
Detection threshold estimation in a single participant using ArOMa-T. (A) Schematic of the numbered peel-and-burst labels on the test card, shaded with gray to indicate differing PEA concentration from lowest (light) to highest (dark); on the actual card, users receive no cues about specific odorant concentration (see Fig. 1). Participants are asked whether they can smell the odor under label 1 (an intermediate concentration). Given a “Yes” response (cyan outline), the app directs them to label 14; a “No” response to this same label (not shown) would send them to panel 6 (a higher concentration that is easier to detect). A “No” response (magenta outline) for label 14 directs the participant to label 11, and so on. The sequence 1, 14, and 11 correspond to 1 possible path through the first 3 questions. (B) Based on psychometric theory and the participant’s responses (outlined in cyan and magenta for “Yes” and “No” responses, respectively, and corresponding to the labels outlined in panel (A)), the algorithm fits a psychometric curve that estimates the probability of a “Yes” response at all concentrations. The solid black line is the point estimate for that curve, and the shaded yellow region is the uncertainty (standard error). The odorant concentration at a “Yes” response probability halfway between the estimated minimum and maximum of that probability is identified as the threshold (vertical dashed line); the uncertainty in this value is indicated with the purple arrow. (C) The same participant completes an additional 5 trials in the same test (for a total of 8 trials); responses are again shown above and below the curve using colored rectangles (cyan square for “Yes,” magenta for “No”; these choices are not indicated in panel (A), as they could take a number of paths). With additional responses, the estimated curve (and threshold) has shifted slightly, and the uncertainty has been substantially reduced. For color figure refer to online version.

The ArOMa-T also employs a unique and novel adaptive threshold estimation algorithm based on a Bayesian model to determine which label the user should peel and sniff next (Fig. 3). Bayesian inference represents a family of approaches for solving problems, and this algorithm is 1 specific instance, although similar approaches have been described elsewhere (e.g. Lesmes et al. 2015; Höchenberger and Ohla 2019a). Such algorithms must be written according to the specific goals, data, and distributional assumptions that correspond to a specific research goal (see below for additional details). In particular, the development of a method that uses a go/no-go task to simultaneously estimate detection threshold and criterion response bias (i.e. false alarm rate) is novel for olfactory testing (and has been described previously in other contexts; e.g. Lesmes et al. 2015). Other reports have used Bayesian approaches with go/no-go tasks for taste thresholds, although the exact model differs (Höchenberger and Ohla 2017, 2019b). Critically, that model does not estimate criterion response bias, while ours does.

Participants are guided through the task with an accompanying app (either locally on smartphone/tablet, or via a web site) that indicates which panel should be peeled and sniffed next. Of labels 1–16, 14 contain different PEA concentrations covering a range of ~3.5 log_10_ units, while 2 of these labels contain no added odorant. Label 0 also has no added odor; it is presented as a reference for the background smell of the label and card. In the version of the ArOMa-T card used here (version 1.P8.1), the positions of the 10 different PEA concentrations are fixed on the card, and there are no cues to participants about the specific concentration found in each numbered label (except for label 0, which is clearly marked “no added odor”).

Before beginning the computer assisted task, participants are asked to sniff label 0 (with no added PEA) to familiarize themselves with the background odor of the card. They start the test by sniffing label 1 (Fig. 3A), where 2 possible responses are considered: “Yes, I can smell it” and “No, I cannot smell it.” All other 15 labels (which may or may not contain an odorant, as some are blanks) are then considered by the algorithm as potential choices for the next trial. The specific label selected by the algorithm to be sniffed next is the concentration (or blank) that is most likely to reduce uncertainty in the running estimate of the detection threshold parameter within the model, weighted by the estimated probability of the “Yes” and “No” responses at the corresponding concentration. This is repeated recursively for all subsequent trials. As more trials are completed, the algorithm fits a psychometric curve that estimates the probability of a “Yes” response at all concentrations (Fig. 3B), and the uncertainty of this curve is substantially reduced with additional trials (Fig. 3C). The algorithm then selects a concentration for the next trial that will be most informative in reducing uncertainty. This differs from a traditional adaptive staircase, where the decision of what concentration to provide next depends solely on the previous 1 or 2 trials (e.g. if a participant gets 2 trials at a given concentration correct, the experimenter provides the next concentration down in the series, while 1 incorrect trial results in 1 concentration level up in the series). In our adaptive algorithm, the next concentration given may or may not be adjacent to the concentration in the prior trial, as the choice of next concentration depends all the prior trials. In the version described here, the test always ends after 8 trials; this number can be adjusted up or down to reflect different tradeoffs between speed and accuracy; alternatively, a stopping criterion based on uncertainty reduction could be used. Because this adaptive model is very computationally demanding (as the complexity doubles with each additional trial), we simulated all possible paths in advance to generate a simple lookup-table that is used in the actual application in real time, using a web browser application on Apple iPads (9.7in screen; Apple Inc., Cupertino, CA). Thus, no internet connection or cloud-based processing was needed to run the algorithm, and test results were stored locally on the iPads. Upon completion, all sensory and demographic data were downloaded for analysis. For this study, only fully deidentified data were accessed.

Here, we used Bayesian inference to estimate the parameters of our psychometric model, applying a weakly informative prior on the detection threshold (log(tau) ~ *N*(−3, 100)) and on the decision criterion (lambda ~ *N*(1, 0.5)). Results summarized below were robust to a wide range of choices for the mean and variance of the prior, including the lack of a prior (i.e. non-Bayesian inference). Theoretical considerations including biological limits on detection threshold motivated our decision to use a weakly informative prior, as did simulated data checks showing more accurate estimation of model parameters and test/retest reliability in synthetic datasets. This is distinct from logistic regression—such as used in other studies (Linschoten et al. 2001; Lötsch et al. 2004; Lawless 2010), which assumes a more rigid probability model for the response.

### Data collection in volunteers at a festival in Twinsburg Ohio

Due to festival logistics and facilities, as well as COVID-19 pandemic-related safety concerns, all testing occurred outside at ambient temperature. Participants were seated at tables under outdoor canopies on an athletic field. Each participant was provided with a physical ArOMa-T card and an iPad with multiple applications, including the custom ArOMa-T web app described above, and a general purpose survey app to gather demographic data. A staff member was available if the participant had questions or technical issues. After consenting, participants entered responses to a few demographic questions, as well as questions on their health history.

Next, participants self-administered the smell test using the physical ArOMa-T card (Fig. 2) and the custom ArOMa-T app described above. Participants were asked to first read the instructions printed directly on the card. They were then instructed to “Please start with Label 0. Peel it back halfway, bring it to your nose, and sniff deeply. Then reseal the label.” The label is hinged at the top, so the participant peels from the bottom upward. Label 0 contains no added odor, so participants can familiarize themselves with the smell of the card and label without PEA. Next, the algorithm directed the participant to label 1, which contains an intermediate concentration of PEA that should be easily perceptible to most normosmic individuals. They were then asked the binary question: Can you smell the scent (YES/NO)? Based on their answer, the algorithm directed the participant to a label containing a lower concentration of PEA (if the answer was YES) or a higher concentration of PEA (if the answer was NO), where they were again asked to peel the label, sniff, and answer the same YES/NO question. This iterative process is illustrated in Fig. 3. After each Y/N question, the algorithm directs the participant to the next label, by number, based on all prior YES/NO responses, which provides a running estimate of detection threshold. Each subsequent trial (and response) improves this estimate (Fig. 3B). Duplication of some concentrations and inclusions of blanks increases the likelihood of faithful responses and allows for criterion bias (e.g. false alarm rate) to be separated from olfactory ability. In the present study, the final detection threshold estimate was determined from a total of 8 trials that include both odorant and blank labels (not including label 0). This design allows for a Bayesian adaptive threshold test, optimized for speedy self-administration and reporting, by selecting the most informative odorant concentration on each trial. The mean time required to complete the test (i.e. all 8 scored questions) was 3.1 min (IQR: 2.2–3.5 min), of which 1.5 min was spent on the instructions (IQR: 0.8–1.7 min).

### Data processing and statistical analysis

Data were analyzed in R using RStudio software (version 2021.09.0). We determined detection threshold estimates in accordance with the ASTM International (formerly American Society for Testing and Materials) Method E679-19 (“Standard Practice for Determination of Odor and Taste Thresholds by a Forced-Choice Ascending Concentration Series Method of Limits”), with minor modifications as described below. Of the 534 participants tested, 66 participants reported “YES” for all PEA containing labels, and “NO” to all blanks they received; their estimated thresholds were imputed with values 1 log unit below the lowest concentration that the ArOMa-T cards presents, in accordance with the standard ASTM E679 decision rule. The specific value used here was −4.5 log_10_ units, and these highly sensitive individuals are shown in a box at the left side of the figure.

At the other extreme, 23 participants reported “NO” for all concentrations presented; again, following the standard ASTM E679 decision rule, these individuals had their estimated threshold set to a value 1 log unit above the highest concentration available on the ArOMa-T card. The specific value used here was 1.0 log_10_ units.

### Testing for age and sex differences in individual thresholds determined with the ArOMa-T

A 2-way fixed model analysis of variance (ANOVA) was performed to determine if estimated odor detection threshold differed by sex or age group. This was followed by a post hoc comparison with Tukey’s HSD (*P* < 0.05) to determine where any group differences occurred.

### Estimation of heritability

Because testing took place at the Twins Day Festival, the sample was enriched in twins and triplets. Of the 534 individuals in the final dataset, 360 were sets of twins (*n* = 180 twin pairs). Of these twin sets, 143 were monozygotic pairs (*n* = 286 individuals) and 37 were dizygotic pairs (*n* = 74 individuals). Falconer’s formula for broad-sense heritability ($H_{b}^{2}=2\times (R_{mz}-R_{dz})$) was used for a heritability estimate. Uncertainty was calculated by applying the Fisher transformation to Pearson correlations and propagating variance through the calculation (Falconer and Mackay 1996). For additional information on heritability and olfaction, see Doty et al. (2011) and Wysocki and Beauchamp (1984). A *z*-test was used to compare the difference between (Fisher *z*-transformed) monozygotic and dizygotic correlation coefficients.

### Exploratory analysis of past COVID-19 status with propensity-matched controls

Given the non-clinical sample, this study was not specifically designed or implemented to compare participants who experienced a past COVID-19 infection with those who did not. However, because 78 participants of the 534 in the final dataset reported a prior case of COVID-19, we felt this incidence (~15%) was sufficient to undertake an unplanned exploratory analysis, with the caveat that no attempt was made to enrich the sample with a prior history of being positive for COVID-19. No data on elapsed time between olfactory testing and COVID-19 illness were collected, so no inferences about speed of recovery should be made from these data.

**Fig. 4.**
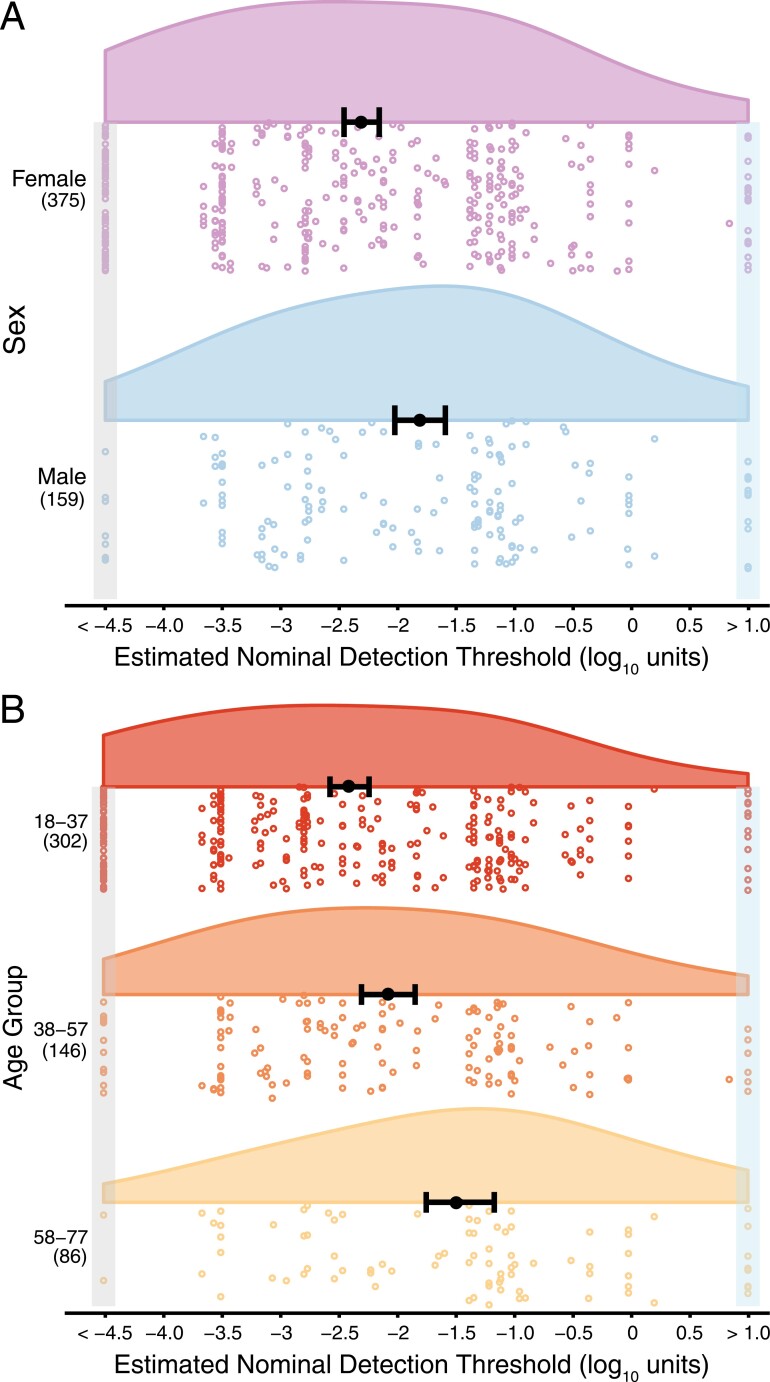
Smell thresholds using the ArOMa-T. (A) Raincloud plots showing odor detection thresholds stratified by sex. Open circles represent odor detection thresholds for individual participants; women are shown in purple and men in blue. (B) Raincloud plots showing odor detection thresholds stratified by age group, with each age group represented by a different color; again, open circles indicate odor detection thresholds for individual participants. In both panels, the solid black dot is the point estimate of the mean and error bars are 95% confidence intervals of that estimate. Group sample sizes are shown in parentheses on the left side of the plot. Thresholds shown in the boxes on the left and right sides were imputed in accordance with the ASTM E679 rules for extreme values outside the range of the concentrations tested; the light blue box on the right highlights functionally anosmic individuals with thresholds above the range tested here, while the gray box on the left indicates highly sensitive normosmic individuals who responded YES to all odorant concentrations. The detection threshold (*x*-axis) is expressed in the base-10 logarithm of the nominal concentrations. For color figure refer to online version.

As ~15% of our sample self-reported a positive history of COVID-19 (*n* = 78), we used propensity matching to identify comparable controls without a positive history of COVID-19. Using the MatchIt package (Stuart et al. 2011) in R, participants were matched based on age, sex, and self-identified racial category, generating 2 equally sized groups of 78 participants for comparison. Due to sex and age group effects in the planned model (Fig. 4), sex and age were included in this exploratory model testing effects of COVID-19 history. However, because of the smaller sample size (*n* = 156, versus 534 in the planned model), age was included in the model as a continuous variable, rather than age group.

A 3-way fixed ANOVA was used to test whether estimated odor detection threshold differed by past COVID-19 status, adjusting for sex and age (as a continuous variable) followed by a post hoc comparison using Tukey’s HSD (*P* < 0.05). In parallel, a 2-sample Kolmogorov–Smirnov (KS) test was also performed to test for differences in distribution shape.

## Results

### Administration of the ArOMa-T

In a non-laboratory setting with an age diverse set of participants, the mean time to complete the ArOMa-T was 2.8 ± 0.9 min, inclusive of time needed to read the instructions (using timestamps built into the software). Individual trials—i.e. peeling a label, sniffing, and answering a single YES/NO question—took an average of 11.6 ± 2.8 s. The false alarm rate for the ArOMa-T (answering “YES” to the first blank presented) was 7.5%, while the rate of answering incorrectly to 2 blanks was 2.6%. For the subset of participants who retook ArOMa-T the following day (*n* = 97), we found a Pearson’s *R* of 0.61, which is comparable to the published test–retest reliability of 0.58 for another rapid card-based measure of olfaction, the NIH Toolbox Odor Identification Test (Toolbox OIT). The Toolbox OIT (Dalton et al. 2013) was used for comparison here because it is also a self-administered, rapid, card-based test that can be mailed (versus other longer tests that require additional materials or a trained administrator). That said, we fully acknowledge that identification and detection are substantially different behavioral tasks (see Introduction).

### Effect of sex and age on thresholds

There was a main effect of sex on odor threshold measured with the ArOMa-T (*F*(1,528) = 15.97; *P* < 0.0001). Female participants showed detection thresholds that were ~0.50 log_10_ units (~3.2×) lower (i.e. more sensitive) than males: −2.31 versus −1.81, respectively Fig. 4B. There was a greater percentage of females (15.5%) than males (5.0%) with the lowest measurable detection threshold (−4.5 log_10_ units; *P* < 0.001, chi-squared contingency test). No significant sex difference was observed at the highest measurable detection threshold (*P* = 0.17).

There was a main effect of age group on estimated odor threshold (*F*(2,528) = 15.32; *P* < 0.0001; Fig. 4B). In Tukey’s HSD (*P* < 0.05), the youngest participants (18–37 years) had lower threshold estimates compared with participants in the oldest group (58–77 years). The size of this mean difference was ~0.94 log_10_ units (a ~8.7× difference in concentration). Participants in the second age bin (38–57 years) had lower threshold estimates compared with participants in the oldest group (58–77 years). The size of this difference was ~0.61 log_10_ units (a ~4.1× difference in concentration). Finally, there was no evidence of an interaction between sex and age group (*F*(2,528) = 0.32; *P* = 0.73).

### Propensity matching and past COVID-19 status

As COVID-19 history had been collected from these participants for another study, we conducted an unplanned, exploratory analysis with participants with a positive history of COVID-19 and controls who were propensity matched in terms of age, sex, and race, to assess whether odor detection thresholds might be elevated in individuals who had previously experienced COVID-19. The ANOVA model testing for an effect of COVID-19 history on smell threshold did not find an association between COVID-19 history and odor detection threshold (*F*(1,152) = 0.11; *P* = 0.74). Specifically, the mean threshold estimate of propensity-matched participants lacking a prior history of COVID-19 was −1.96 ± 1.46, compared with −1.88 ± 1.30 for participants who self-reported a prior COVID-19 infection, a nominal difference of ~0.08 log units (~1.2× difference). Likewise, we found no evidence for differences in threshold distributions between those who previously had COVID-19 and those who did not (KS test statistic = 0.09; *P* = 0.91). Collectively, these exploratory analyses provide no evidence that odor thresholds are elevated in a convenience sample of individuals who have previously recovered from acute COVID-19. However, this finding is only tentative and should be confirmed in larger samples with specific study recruitment intended for such comparisons.

### Heritability of detection thresholds measured with the ArOMa-T

The large number of both monozygotic and dizygotic twins in our sample offered a unique opportunity to conduct a preliminary estimate of heritability for detection thresholds collected using the ArOMa-T. Dizygotic twins exhibited weak correlation in estimated detection thresholds (*R* = 0.19 ± 0.16), while monozygotic twins showed a slightly stronger correlation (*R* = 0.46 ± 0.07; *P* = 0.05). Using Falconer’s formula (see Methods), we estimate a broad-sense heritability of *H*^2^ = 0.55 ± 0.36.

## Discussion

The primary goal of this study was to assess the feasibility of a novel, card-based smell test, the ArOMa-T, for determining odor detection thresholds. While odor detection threshold estimates depend on the exact psychophysical method used to operationally measure threshold (Leek 2001; Lawless 2010), threshold-based assessments have some advantages over other measures of olfactory function as they avoid issues of prior familiarity, memory recall, and naming ability. However, threshold-based methods can be tedious and slow, so they are often avoided in favor of more rapid methods when time is at a premium. For example, during the planning phase of the NHANES chemosensory examination (e.g. Hoffman et al. 2016; Gallo et al. 2020), threshold methods were omitted from the testing battery due to the time required.

### Novelty of the ArOMa-T

The COVID-19 pandemic motivated us to develop the ArOMa-T to address the unmet need for a rapid, disposable test able to measure odor thresholds in a wide variety of settings. We did so by integrating existing technology into a unique and novel test platform. For example, peel-and-burst odor delivery systems have been used in other smell tests like SCENTinel (Parma et al. 2021), but we exploited the resealability of this specific peel-and-burst system to allow us to terminate odor delivery. Other card-based tests of odor identification use scratch and sniff encapsulation (e.g. mPST) or burst labels that cannot be closed once opened (Okutani et al. 2013). Elsewhere, Sniffin’ Sticks uses a classical adaptive staircase method to assess odor thresholds, but the original algorithm is not Bayesian. After present data were collected, a modified Sniffin’ Sticks protocol that uses a Bayesian QUEST algorithm was published (D’Alessandro et al. 2022); however, the odor wands used by Sniffin’ Sticks are neither mailable, portable, or disposable, and the test still requires a test administrator. Further, even the Bayesian QUEST version of Sniffin’ Sticks still uses an odor triplet (i.e. a triangle test), which is slower than the go/no-go task used here. Thus, the ArOMa-T is unique in that it combines the resealable labels, the self-guided app, a bespoke algorithm, and the physical card to create a novel threshold test that is (i) rapid, (ii) disposable, (iii) portable, and (iv) mail compatible.

### Administration of the ArOMa-T

Here, we found the ArOMa-T is a fast, easy to use, field-deployable test. Over 500 participants were able to complete the test despite its being administered in an outdoor, festival setting. The median time to complete an individual ArOMa-T—under 3 min—compares favorably to commercially available smell tests like the UPSIT and Sniffin’ Sticks, which can take 8 or more minutes to complete. When participants were presented with 2 blanks (versus 1), the false alarm rate—the fraction of participants who responded YES to all blanks—was dramatically reduced from 7.5% to 2.6%. In future studies or in clinical use, it will be trivial to adjust the algorithm (e.g. multiple blanks, more total trials). Still, the test–retest reliability of ArOMa-T in this study (Pearson’s *R* of 0.61) is comparable to other validated self-administered rapid smell tests, such as the NIH Toolbox OIT, which has a test–retest reliability of 0.58 (Dalton et al. 2013). The Pearson’s *R* observed here for the ArOMa-T suggests this test is both reliable (i.e. it provides an accurate representation of a participant’s performance across testing sessions) and internally valid. Elsewhere, Croy et al. reported stronger test–retest correlations for PEA thresholds in normosmic individuals: specifically, when using a 2-down-1-up staircase protocol with 8 or 16 PEA concentrations (i.e. wide step versus narrow step), the test–retest correlation was ~0.84 (Croy et al. 2009). However, this adaptive staircase protocol required presentation of 16–20 triplets on average to reach the stopping point, with a mean testing time in normosmic individuals of 6–8 min. Whether the ArOMa-T could be optimized to achieve comparable reliability by adding more trials remains to be determined. Additional work is also needed to directly compare both total test time and test–retest reliability of the ArOMa-T to other threshold methods such as Sniffin’ Sticks (Kobal et al. 1996) or Snap & Sniff (Doty et al. 2019).

### Effects of sex on olfactory threshold detection

This study recapitulated well-known sex differences in odor thresholds where females have greater olfactory sensitivity relative to males (Fig. 4) (Hedner et al. 2010; Oleszkiewicz et al. 2019; Sorokowski et al. 2019). Females have consistently been shown to be more sensitive than males for many odorants, including 1-butanol, 1-hexanol, and 1-octanol (Koelega and Köster 1974; Cometto-Muñiz and Abraham 2008). While there is no definitive explanation for the consistent observation that females show superior olfactory performance than males, interactions of sex hormones with the olfactory system have been postulated (Koelega and Köster 1974; La Mantia et al. 2001; Doty and Cameron 2009). Separately, females may have superior ability to pick up odors from a multitude of external stimuli, which has been termed odor awareness (Herz and Inzlicht 2002). Last, it has been suggested the rate of olfactory decline is greater among males (Pinto et al. 2015) and males may be more prone to harmful occupational exposure to toxic compounds that damage the olfactory system (Corwin et al. 1995). Regardless of the underlying reasons for these commonly observed sex differences, it is clear that the ArOMa-T has sufficient sensitivity to discern such expected sex differences.

### Effects of age on olfactory threshold detection

We also found that mean odor detection thresholds increased with age (Fig. 4), similar to prior work (Hedner et al. 2010; Oleszkiewicz et al. 2019). Notably, younger participants in our study were more likely to exhibit the lowest odor detection thresholds. The higher average odor detection thresholds seen in the oldest participants for this study is consistent with previous studies that found about half of the US population between the ages of 65–80 experience smell loss, and this prevalence increases to about 3 quarters of those over the age of 80 (e.g. Murphy et al. 2002). A relationship between age and olfactory decline has been seen when odor identification ability was assessed by itself (Doty 1989), or when odor identification, discrimination, and detection threshold were all tested (Oleszkiewicz et al. 2019). Indeed, odor identification and threshold tests are both sensitive to age-related smell loss (Cain and Stevens 1989; Schubert et al. 2017), consistent with present data gathered with the ArOMa-T.

### Effects of past COVID-19 status on olfactory threshold detection

Multiple studies have explored olfactory detection thresholds as a measure of smell loss due to current or prior SARS-CoV-2 infection (e.g. Le Bon et al. 2020; Vaira et al. 2020). One found that odor detection threshold scores were more affected than discrimination and identification scores in COVID-19 patients tested with Sniffin’ Sticks at least 2 weeks after symptom onset (Le Bon et al. 2020). Elsewhere, it was reported that the majority of COVID-19 patients had impaired olfactory thresholds when tested with the Connecticut Chemosensory Clinical Research Center orthonasal olfaction test approximately 2 weeks after a positive SARS-CoV-2 PCR test (Vaira et al. 2020). Further, it has been reported that olfactory threshold scores from Sniffin’ Sticks were more affected than scores for odor identification and discrimination in hospitalized patients with COVID-19 tested ~1 month after diagnosis (Iannuzzi et al. 2020). Collectively, these reports suggest detection threshold may be particularly susceptible to COVID-19; if confirmed, this might suggest some individuals with COVID-19 experience hyposmia that is missed with an odor identification task.

The relationship between subjective assessment of smell and controlled psychophysical testing remains highly contentious (see Ekström et al. 2018). Self-report can be subject to recall bias, and many with measurable loss may be unaware of this loss (Murphy et al. 2002; White and Kurtz 2003). On the other hand, measures of subjective loss or dysfunction may better capture quality of life issues, including dietary intake (Fluitman et al. 2021). Notably, qualitative disorders like parosmia and phantosmia can only be assessed via patient history and self-report, as no objective tests exist for these conditions (Parma and Boesveldt 2021). Our results conflict somewhat with some prior work, as we saw no convincing evidence that thresholds were elevated in those who had recovered from COVID-19. However, this was a small convenience sample of individuals without active COVID-19, and we have no estimate of the elapsed time between illness and olfactory testing. Additional work in larger cohorts with recruitment stratified by current COVID-19 status and/or past history is warranted to resolve these questions. The ArOMa-T may be especially well suited for such study designs, given that it is mailable, rapid, and suitable for field use.

## Limitations

Several limitations should be noted. Foremost, the sample was predominantly non-Hispanic White, so results may not generalize to other demographic groups. Also, the test setting could have biased recruitment of older individuals toward those in exceptionally good health—i.e. a “healthy worker effect” (McMichael 1976). This may partially explain why some participants in the 58–77 age category show the same or a lower olfactory detection threshold as those in the 18–37 age category. Thus, we cannot make sweeping generalizations about aging from these data. Further, our heritability estimate is highly uncertain, as the number of twin pairs is too small for a more precise estimate. The moderate heritability observed here may reflect numerous causes of differences in overall olfactory ability including differences in specific anosmias, nasal patency, or myriad other factors that are shared by monozygotic siblings. While PEA is commonly used in olfactory testing (e.g. Sniffin’ Sticks), we cannot exclude that performance on this test may vary as a function of individual genetics (Hsieh et al. 2017). Because specific anosmias for individual odors can arise from genetic variation (Trimmer et al. 2019), a diagnosis of generalized anosmia cannot and should not be made with a single odorant. Rather, follow-up testing with additional odorants would be needed before a formal diagnosis of anosmia could be made clinically. Separately, while we do not have any evidence of cross contamination between labels, we cannot entirely rule out this possibility. However, the test was administered outside where airflow presumably mitigated any residual odors once labels were resealed. Additional work in larger and more deliberately stratified samples will be necessary to resolve these questions, as well as questions of clinical utility and usability. Finally, while our test is deliberately designed to separate response bias from underlying ability, a deliberate bias (such as malingering) would be difficult to distinguish from anosmia without additional assessments.

## Conclusions

We found that the ArOMa-T can be used as a rapid olfactory screening test to capture variation in detection thresholds. We observed differences in estimated detection thresholds between sex and age groups, highlighting the ability of the ArOMa-T to reproduce expected population level findings, as well as demonstrating its ability serve as a portable and rapid smell test. This is highly advantageous compared with other olfactory tests that take longer to complete, have portability limitations, or must be administered by a trained individual. In a field-based convenience sample, no evidence was found to suggest detection thresholds differ between participants who report a history of COVID-19 and matched controls who did not. However, these tentative null results require confirmation in a study specifically designed to explore this question. Collectively, our results suggest the ArOMa-T is able to reproduce sex and age effects previously observed with more time-intensive testing methods.

## Data Availability

The full data underlying this article cannot be shared publicly to protect the privacy of individuals that participated in the study. Deidentified data will be shared on reasonable request to the corresponding author.
